# ABO Blood Groups and Cardiovascular Diseases

**DOI:** 10.1155/2012/641917

**Published:** 2012-10-22

**Authors:** Hanrui Zhang, Ciarán J. Mooney, Muredach P. Reilly

**Affiliations:** ^1^Cardiovascular Institute, Perelman School of Medicine, University of Pennsylvania, Philadelphia, PA 19104-6160, USA; ^2^Institute for Translational Medicine and Therapeutics, University of Pennsylvania School of Medicine, Philadelphia, PA 19104-5158, USA

## Abstract

ABO blood groups have been associated with various disease phenotypes, particularly cardiovascular diseases. Cardiovascular diseases are the most common causes of death in developed countries and their prevalence rate is rapidly growing in developing countries. There have been substantial historical associations between non-O blood group status and an increase in some cardiovascular disorders. Recent GWASs have identified ABO as a locus for thrombosis, myocardial infarction, and multiple cardiovascular risk biomarkers, refocusing attention on mechanisms and potential for clinical advances. As we highlight in this paper, more recent work is beginning to probe the molecular basis of the disease associations observed in these observational studies. Advances in our understanding of the physiologic importance of various endothelial and platelet-derived circulating glycoproteins are elucidating the mechanisms through which the ABO blood group may determine overall cardiovascular disease risk. The role of blood group antigens in the pathogenesis of various cardiovascular disorders remains a fascinating subject with potential to lead to novel therapeutics and prognostics and to reduce the global burden of cardiovascular diseases.

## 1. Introduction

In 1901, Landsteiner identified ABO blood groups as the first recognized human blood group system. The clinical significance of ABO blood type extends beyond transfusion medicine and solid organ/hematopoietic transplantation. To date, numerous reports have suggested important associations between ABO blood groups and various diseases, for example, gastric cancer [1], periodontal diseases [2], and cardiometabolic diseases [3, 4].

According to World Health Organization (WHO) data, cardiovascular diseases (CVDs) are and will remain the leading causes of death globally: an estimated 17.3 million people died from CVD in 2008, representing 30% of all global deaths (WHO Media Centre (2011), cardiovascular diseases (fact sheet). retrieved from http://www.who.int/mediacentre/factsheets/fs317/en/index.html). Studies on the associations between CVD and ABO blood groups have a long history. In 1955, Woolf proposed an odds ratio as a measure to quantify the disease risk conferred by blood group type [5]. In 1969, Jick et al. reported a deficit of patients with blood group O among those who received anticoagulants for venous thromboembolism [6]. Prior to mutation detection in haemophilia carriership analysis, likelihood ratios of carriership of hemophilia A were based on Factor VIII levels conditional on blood group [7]. A number of later studies elucidated that ABO blood groups, particularly non-O blood groups, are associated with major cardiovascular risk factors and/or increased rate of cardiovascular events [8–13]. However, there is limited consensus regarding the magnitude and significance of the ABO effects at the population level, and whether it relates to all disorders equally or predominantly modulates thrombotic pathways and disorders [14]. This paper summarizes the basic concepts of the biochemistry of ABO blood groups and recent findings of their relations to CVD.

## 2. Biochemistry and Population Distribution of ABO Blood Groups

The ABO blood group is determined by the presence of A and B antigens on the surface of the red blood cells (RBCs). In addition to RBCs, these antigens are widely expressed on the membranes of a wide variety of cells, including platelets, vascular endothelium and epithelium [15] as well as in saliva and body fluids [16]. The biochemistry of the ABO blood group system has been reviewed recently [16]. Briefly, the ABH blood group antigens consist of terminal carbohydrate molecules which are synthesized by the sequential action of the ABO glycosyltransferases. The *ABO* glycotransferase (transferase A, alpha 1,3-N-acetylgalactosaminyltransferase; transferase B, alpha 1,3-galactosyltransferase) gene encodes proteins related to the ABO blood group system [17, 18]. The active ABO glycotransferases catalyze the addition of specific monosaccharides to a common core precursor antigen (H) to form distinct A and B antigens. Individuals with blood group O express only the basic H antigen [19] due to a deletion of guanine-258 in the region of the gene encoding the N-terminus of the protein which results in a frameshift and translation of a protein lacking glycosyltransferase activity [17, 18].

The frequency of the common ABO phenotypes varies among different populations. Populations with a high frequency of the A phenotype are found mainly in Northern and Central Europe [18]. The B phenotype is most frequent in Central Asia [18]. Blood group O is the most frequent phenotype globally, with parts of Africa and Australia showing highest frequencies [18]. The reasons for the observed differences among populations are not well understood, although several theories have been proposed. Evolutionary selection based on pathogen-driven blood group antigen changes may be one of the major contributors [18]. Under this theory, terminal carbohydrate modification on host proteins, lipids, and cells plays a significant role in modulating interactions with pathogens. Thus, ambient pathogens are thought to have driven the regional evolution and selection of host blood group antigens that provide survival advantage to distinct geographic pathogen exposures. 

## 3. Genome-Wide Association Studies Confirmed ABO as a Locus for Venous Thromboembolism and Myocardial Infarction

The widespread use of genome-wide association studies (GWASs) over the last 5 years has spurred an enormous acceleration in discoveries across the entire spectrum of CVD [20]. Recent GWASs have confirmed *ABO* as a locus for venous thromboembolism (VTE), myocardial infarction (MI), and multiple cardiovascular biomarkers (Table 1).

One of the most studied aspects of the *ABO* gene is its relationship with von Willebrand factor (VWF) [17, 36, 37]. In 2003, a family-based linkage screen was carried out to determine the loci involved in VWF variation in 398 Spanish individuals. Markers at the chromosome 9q *ABO* locus region harbored the highest LOD value of 3.46 [38]. Subsequently, several GWASs have shown that carriers of single nucleotide polymorphisms (SNPs) that mark non-O blood group types have higher levels of plasma VWF when compared to O individuals. A recent GWAS of 7856 European participants in the Atherosclerosis Risk In Communities (ARIC) cohort study showed that ABO blood group O carriers had a 25% average reduction in plasma VWF levels when compared with non-O blood group carriers. SNP rs514659, which was used to tag the O blood type, contributed to 15.4% of circulating VWF variance [33]. Additionally, a genome-wide meta-analysis of 4 cohorts found that rs657152, which also tags the blood group O, was strongly associated with circulating VWF levels and that blood group O individuals had 22–30% lower plasma VWF when compared to non-blood group O individuals [28]. A relationship between Factor VIII (FVIII) plasma concentrations and ABO blood groups has also been seen. However, in a Cohorts for Heart and Aging Research in Genome Epidemiology (CHARGE) Consortium GWAS, no unique genetic variants within the ABO locus were found to affect FVIII independently of VWF [34]. As VWF binds and transports FVIII, the correlation between the ABO gene and FVIII is most likely mediated via VWF [39]. 

As increased plasma levels of VWF and Factor VIII are associated with greater risk of thrombosis [40, 41], many studies have examined the connection between ABO blood group and thrombotic risk. In a GWAS published in 2009, SNPs rs8176750, rs8176746 and rs8176719, which tag the A2, B, and O ABO blood groups, respectively, showed that genetically inferred blood type O had 67% lower risk of VTE than non-O blood groups. Additionally, the A2 blood group had 47% lower risk of VTE when compared to the other non-O blood group phenotypes [32]. Blood type A2 was also shown to be associated with lower VTE risk in a recently published GWAS involving 1,503 VTE patients in which rs8176704 was used to tag the A2 blood group [23]. These data suggest that the decreased risk of VTE is a result of reduced H antigen glycosylation, as the A2 allele contains a 1061delC that results in the synthesis of an enzyme that has 30–50 fold less A transferase activity than the A1 allele product [42]. Blood group genotypes may be more informative than blood group phenotypes in studying the association between blood groups and VTE since genotypes can distinguish between heterozygous and homozygous carriers of A, B, and O alleles and between A1 and A2 alleles [43, 44]. One GWAS found that the A11 allele, tagged by rs529565 and rs657152, and the B allele, tagged by rs8176749, were associated with 56% and 16% increased risk of VTE, respectively, when compared to the O11 allele. Moreover, when compared with carriers of the O1O1 diplotype, VTE risk was increased by 79% for the A11 diplotype, 82% for the B diplotype, and 170% for the AB diplotype carriers. Overall, non-O categories combined were associated with a 77% increased risk of VTE when compared to the O category [29]. The effect of ABO genotype on thrombosis risk was also investigated in a case-control study of 471 patients and 471 controls of the Leiden Thrombophilia Study (LETS) which revealed that non-OO genotypes, except homozygous A2 and A2-O combinations, were associated with increased thrombotic risk when compared to OO genotypes. The relative thrombotic risk of AB genotypes and A1-combinations was increased by 90–110% when compared to OO genotypes and the relative thrombotic risk of the homozygous B genotype and B-O combinations was increased by 60% [45].

The ABO locus has also been associated with arterial thrombosis in studies of MI. Our group reported that all 11 SNPs that exceeded genome-wide significance for MI in patients with established coronary atherosclerosis mapped to the ABO locus. The risk alleles at rs514659 (odds ratio 1.21; *P* = 7.62 × 10^−9^) and rs687289 (odds ratio 1.19; *P* = 7.75 × 10^−9^) were perfect tags for the loss of function ABO O blood group demonstrating that functional ABO glycotransferases conferred increased risk of MI. Further analysis found that rs514659 was associated with coronary artery diseases (CADs) when complicated by MI but not with CAD without MI, suggesting that the primary relationship of ABO to clinical CAD is through modulation of coronary thrombosis or plaque rupture in patients with established coronary atherosclerosis rather than through primary promotion of atherosclerosis per se. 

The increase in MI risk for non-O blood type individuals has been suggested for some time through epidemiological studies, although there has been debate as to which ABO blood group phenotypes confer the largest increase in risk [8, 11, 46–49]. Just recently, He et al. reported results of two large prospective studies of incident coronary heart disease (CHD) as well as a meta-analysis of all prospective data [50]. The Nurses' Health Study (including 62,073 women ages 30 to 55 at baseline) and the Health Professionals Follow-up Study (including 27,428 men ages 40 to 75 at baseline) were followed up to 2006 and recorded 2,055 cases of CHD in the two cohorts. Individuals with self-reported non-O blood type had an age-adjusted hazard ratio (HR) of 1.09 (95% CI 1.03 to 1.17, *P* = 0.005) for risk of developing CHD. Associations between blood type and CHD risk were not modified by age, physical activity, alcohol consumption, smoking status, or diabetes history. A meta-analysis of an additional six prior cohorts, for a combined total of 114,648 individuals and 5,741 CHD cases, also showed a significant pooled relative risk for CHD in patients with non-O blood type of 1.11 (95% CI 1.05 to 1.18, *P* = 0.001). Among participants in the cohort, those with type O blood were significantly less likely to develop CHD when compared against types B (HR 1.11, 95% CI 1.01 to 1.23) and AB (HR 1.23, 95% CI 1.10 to 1.37), with a trend toward a higher risk for patients with type A blood (HR 1.05, 95% CI 0.98 to 1.13).

It is plausible that ABO modulation of VWF-related thrombosis accounts for the ABO association with MI. However, ABO antigens are expressed also on distinct platelet proteins, including GPIIb, a subunit of the fibrinogen receptor heterodimer [51–54], and may therefore modulate specific platelet functions in arterial thrombosis and MI (see below). In addition, ABO modulation of atherosclerotic plaque rupture and atherosclerosis itself cannot be discounted without further study. 

## 4. Associations of the ABO Locus with Markers of Endothelial Function and Serum Lipoproteins

The ABO glycotransferase may have broader impact on atherosclerotic CVD than simply through modulation of thrombosis. A series of GWAS have linked the ABO locus to circulating levels of soluble intercellular adhesion molecule-1 (sICAM-1), soluble P-selectin (sP-selectin), and soluble E-selectin (sE-selectin). Notably, mechanistic studies in rodent models have implicated these proteins in atherosclerosis [55–59] and their blood levels in humans correlate with increased risk of CVD events [60, 61]. Paré et al. showed that SNP rs507666, located in Intron 1 of the *ABO* gene and a perfect tag for ABO blood group A1, was associated with decreased levels of sICAM-1 (*P* = 5.1 × 10^−29^) when compared to the O allele and contributed to 1.5% of the total sICAM-1 concentration variance [31]. The same group confirmed this relationship between rs507666 and circulating sICAM-1 levels in a larger GWAS published in 2011 [30]. Blood levels of sP-selectin and sE-selectin are also associated with SNPs in the ABO region [21, 22, 25]. A recent meta-analysis showed that, compared with major allele homozygotes, heterozygote and minor allele homozygote individuals for the rs507666, rs579459, and rs651007 SNPs, which demonstrate a high degree of linkage disequilibrium with each other (*r* ^2^ ~ 0.96) and tag the ABO A1 subtype, had lower plasma levels of sICAM-1, sP-selectin, and sE-selectin [27]. A recent GWAS has also identified a potential relationship between ABO and circulating levels of angiotensin-converting enzyme (ACE); when compared to the ABO blood group O, mean ACE activity in carriers of blood group B was significantly increased (*P* = 2.3 × 10^−10^) while ACE activity in blood group A was decreased (*P* = 1.5 × 10^−8^) [24]. 

Past epidemiological studies, some dating as far back as 50 years ago, have suggested evidence for ABO association with circulating levels of cholesterol, with non-O groups appearing to have higher levels [47, 62–65]. For example, in 1976, Garrison et al. published an epidemiological analysis in the Framingham Heart Study showing consistent elevations of serum cholesterol levels in non-O blood groups when compared to the O blood group [47]. Some recent GWASs and their meta-analyses support this potential role for ABO genotypes in modulating circulating levels of total and LDL cholesterol, as well as phytosterols, established causal risk factors for atherosclerotic heart diseases [26, 35, 66]. A meta-analysis of 46 lipid based GWAS reported an association between *ABO *SNPs and serum cholesterol levels. Total cholesterol was increased by 2.3 mg/dL in heterozygote individuals for the *ABO *rs651007 SNP when compared to major allele homozygotes (*P* = 8.66 × 10^−21^), while LDL cholesterol was increased by 2.05 mg/dL in heterozygote individuals for the *ABO *rs649129 SNP when compared to major allele homozygotes (*P* = 7.85 × 10^−22^) [26]. A GWAS published in 2010 found that the ABO locus showed genome-wide-significance for association with phytosterol levels. Specifically, rs657152, which tightly tags the O1 allele, was found to be associated with decreased levels of circulating phytosterols. In a separate analysis, the study reported that individuals with the O allele had decreased campesterol concentrations when compared to the A and B alleles. Furthermore, additional analyses revealed that rs657152 was associated with reduced CAD risk (*P* = 4.0 × 10^−5^) when compared to alleles that were associated with increased phytosterols [35]. These epidemiological and genetic associations of ABO blood type with levels of circulating lipoproteins and sterols underscore the need for functional studies that define the mechanistic basis of these relationships and the potential for therapeutic translation. 

Overall, these pleiotropic associations with cardiovascular risk biomarkers suggest a complex role of ABO in atherosclerotic and vascular diseases with distinct ABO genotypes and ABO functions contributing to multiple causal pathways, for example, ABO genotypes related to blood group O and loss of glycotransferase function protect against VTE and MI while distinct genotypes relating to specific A and B blood subgroups and glycotransferase functions may have a more subtle and distinct impact on endothelial function, lipoproteins, and atherosclerosis (Table 1). 

One paradox that has limited clinical interpretation of GWAS discoveries, including that for the *ABO* locus, relates to the highly significant *P* values reported for common disease variants despite very small effect sizes (e.g., odds ratio). Unlike rare disease causing mutations, common variants for complex diseases have either minor functional effects or are simply crude markers for rare functional variants; their strength of association with disease is often very small despite highly significant *P* values (which relate mostly to the sample size of GWAS). However, the weak effect sizes of these variants should not be mistaken for a lack of clinical importance of a particular gene (or protein/biomarker) or its therapeutic manipulation in a complex disease process. For example, rare variants in 3-hydroxy-3-methyl-glutaryl-CoA (HMG-CoA) reductase have quite weak associations with LDL cholesterol (LDL-C) levels and are well down the list of top GWAS findings for LDL-C [26]. Thus, rather than attempting to infer clinical relevance based on the strength of associations, the key importance of GWAS is simply to identify novel genes and proteins for human disease that warrant further study to establish their mechanistic role and clinical importance. Over time, the totality of evidence derived from human, animal, and functional studies allows an interpretation of the clinical and therapeutic importance of the initial significance level for a locus in a GWAS, the magnitude of effect on a protein level, and the relative risk of a given protein level for a disease phenotype. For the *ABO* locus, further mechanistic and functional studies are required in order to define the clinical and therapeutic possibilities. 

## 5. Potential Mechanisms for the Association between ABO and CVD

Following recent GWAS discoveries, there is a resurgent interest in identifying the mechanistic links underlying the association of the ABO locus and glycotransferase functions with CVD. Most attention until recently was focused on the role of ABO in regulating VWF bioactivity and related thrombotic pathways, but emerging work is defining more broadly the role of ABO glycotransferase activity in modulating multiple endothelial, platelet, and cardiometabolic pathways and exploring whether these effects are causally involved in cardiometabolic diseases. 

### 5.1. VWF and FVIII as Candidate Mechanisms

VWF and FVIII are glycoproteins (GPs) that circulate together in normal plasma as a noncovalent complex and both play important roles in normal hemostasis. VWF is a carrier for FVIII and protects it from inactivation. VWF recruits platelets to the site of clot formation during primary hemostasis. Factor VIII is released from VWF by the action of thrombin and participates in the coagulation cascade. Thus, both VWF and FVIII are key proteins in the formation of occlusive thrombi in injured vessels [67]. As described in preceding sections, relative to non-group O, carriers of ABO blood group O have significantly lower circulating plasma VWF and FVIII levels [68]. Although this clinically important effect of ABO group on plasma VWF-FVIII levels is well established, the mechanism through which it is mediated is not completely resolved. ABO appears to have direct functional effects on circulating VWF and indirectly (via influence of VWF levels) modulates FVIII levels. The presence of abundant ABH carbohydrate molecules on the VWF oligosaccharide side chains provides the mechanistic basis for ABO regulation of VWF levels. The active ABO A and B glycotransferase enzymes, found in Golgi of endothelial cells, generate terminal carbohydrate modifications, A and B antigens, on the existing VWF “H” oligisacharides, whereas the enzymatically inactive ABO O protein cannot modify these VWF H antigens. The addition of A or B terminal carbohydrate antigens to VWF in endothelial cells might influence circulating VWF levels and function by several mechanisms: altering the rate of VWF synthesis and/or secretion, regulating VWF proteolysis induced by its major protease, ADAMTS13, modulating VWF clearance, or changing VWF biological activity: or perhaps some combination of these events [17]. A number of studies suggest that it is unlikely that ABO effects on VWF levels are mediated by alterations in the biosynthesis and secretion of VWF [17]. In contrast, it has been demonstrated that the activity of ADAMTS13 differs against VWF of different blood groups, with both the level of ADAMTS13 activity [69], and the rate of VWF proteolysis by ADAMTS13 [70], being higher in blood group O as compared to non-O individuals. Thus, the absence of VWF terminal carbohydrate modifications in individuals with ABO blood group O increases the susceptibility to and rate of proteolysis by ADAMTS13, although data available so far are not in support of the role of proteolysis by ADAMTS13 in VWF clearance from the circulation [71]. Due to their physical association in blood, lower circulating VWF results in lower levels of FVIII. Whether VWF in platelets (a relatively abundant source) undergoes any modification by ABO remains controversial; such modification could alter platelet production and subsequent turnover of VWF, particularly locally during platelet-driven arterial thrombosis, although this remains to be established. Indeed, current knowledge suggests that ABO does not modify platelet VWF [72], but the role of ABO in regulating platelet VWF and platelet function in thrombosis requires greater study (see below). 

### 5.2. Other Endothelial Molecules and Platelet Proteins as Potential Mediators

Soluble levels of multiple adhesion molecules, mostly derived from endothelial cells and platelets, have been associated with coronary heart disease [61], and other cardiovascular conditions [73]. In large-scale genomic studies, plasma levels of sP-selectin and sICAM-1 were associated with ABO gene variants, but no association was found between platelet-bound P-selectin levels and ABO blood group, suggesting that the ABO blood group may influence proteolysis and clearance from the circulation, rather than its production and cellular presentation [25]. However, the specific mechanisms of ABO regulation of circulating levels of these endothelial GPs are largely unknown. Further, the implications for their function in atherosclerosis and clinical CVD require elucidation. 

Blood group ABO antigens are known to be carried by several platelet GPs, for example, GPIb, GPIIb, GPIIIa, and platelet endothelial cell adhesion molecule (PECAM) [53], that play important roles in platelet function. Platelet ABH expression is a stable, donor-specific characteristic with 5% of A1 donors typing as either ABH high- or low-expressers [74], with high levels of A antigen on various GPs from high-expresser platelets, especially GPIIb and PECAM (CD31) [51]. GPIIb is an integral component of the GPIIb-GPIIIa fibrinogen receptor complex, which represents the critical final common pathway for platelet-driven thrombosis in hemostasis and pathologic arterial thrombosis including acute MI. Genetic variation in GPIIb that modulates fibrinogen binding has been associated with altered risk of thrombosis and MI [75–77], so it is conceivable that ABO-driven carbohydrate modification of GPIIb might alter its functional interactions with fibrinogen and thus platelet-mediated thrombosis. However, this hypothesis has not been adequately addressed to date. Besides GPIIb and PECAM, blood group A antigen is also expressed on other uncharacterized platelet proteins (70–90 kDa) having electrophoretic mobilities closely resembling those of GPIV and GPV [52]. Thus these and other uncharacterized ABO-expressing platelet proteins may also act as potential functional modulators of the ABO associations with arterial thrombosis and cardiovascular events.

The list of GPs carrying blood type ABO antigens is growing. Some of the most abundantly expressed GPs, such as PECAM and VWF, represent major proteins recognized to carry ABO antigens. However, there is a great need to identify all ABO target proteins and glycolipids in order to understand the nature and/or properties of GPs carrying ABO antigens and to reveal novel, potentially cell-specific, mediators of CVD.

## 6. Perspectives

Despite the relative simplicity of the A and B antigens, especially considering the modest biochemical difference between them, the ABO blood group system appears to be one of the most interesting, both clinically and scientifically. It divides the world's population into four major groups. The common frequency of each major blood group, especially blood group O (the loss of glycotransferase function ABO type), suggests important evolutionary selection pressures distinct from heart disease which now have subtle but significant impact at the population level on complex diseases in modern society. Much larger epidemiological and genetic studies are required to determine risks of CVD with particular major and minor ABO blood group. Such human data will inform translational and mechanistic studies by pointing to specific ABO glycan modifications that appear to be the drivers of increased or decreased risk. However, such studies are inherently limited because they fail to illuminate the specific cell/tissue where glycan modifications are mediating their actions and do not define the proteins or lipids carrying such modifications that are functionally altered and mediate disease. 

In future studies, it is of critical importance to establish clear mechanistic basis for the association between ABO blood groups and CVD. Advances in our understanding of the physiologic importance of the glycan structures of VWF have provided a model and elucidated one mechanism, possibly the major one, through which ABO blood group determines risk of thrombosis and acute cardiovascular events. However, there are several other known endothelial and platelet GPs, discussed above, that are potentially functionally modified by ABO and candidate causal mediators in atherosclerosis and CVD. How and where these proteins are modified and whether their carbohydrate alterations have functional impact on disease requires further study. The increased risks associated with non-O blood groups might be attributed to higher levels or functional modification of specific endothelial-derived GPs, specific platelet GPs, both sources, and/or GPs from additional cells and tissues although leukocytes are not likely primary sources as they lack the ABO enzyme [78]. 

The study of glycobiology in complex disease is in its relative infancy. Unbiased approaches are required to discover and identify the population of proteins and lipids that are modified by ABO (and other glycotransferases), in cells and tissues of specific relevance to atherothrombosis, so that we can define all potential mediators of specific aspects of risk and specific disease manifestations. Technological and bioinformatics advances, including mass-spectrometric approaches, are beginning to emerge which will permit such unbiased work. The tissue-specific expression and function of ABO and many known glycotransferases [79] suggest strongly that their actions and targets will be regulated at a cell- and tissue- specific levels coincident with roles in paracrine- and cell-specific events in atherothrombosis. A naïve impression might suggest that inhibition of ABO glycotransferase functions, akin to the naturally occurring ABO O blood group, might provide a therapeutic strategy for lowering the risk of thrombosis and CVD. However, such a pharmacological approach would likely have untoward hematological and immunological consequences and/or nonspecific off target actions in cells and tissues not relevant to vascular diseases. Identification of the specific protein and lipid targets of ABO which mediate the functional effects in thrombosis, atherosclerosis and CVD is likely to provide greater opportunity for targeted therapeutic development and clinical translation. Whether targeting VWF carbohydrate modifications might provide clinically meaningful opportunities for treatment of thrombosis and MI is an open question. 

There is much to be done to understand the role of ABO and glycobiology in CVD, and the next decade should see many advances in the basic biology, mechanistic actions, and diagnostic, prognostic, and therapeutic possibilities in humans. Thus, future studies to further define the association between ABO blood groups and cardiovascular events and risks, and to elucidate biochemical mechanisms responsible for these associations, are not only of basic scientific interest but also of translational clinical importance.

## Figures and Tables

**Table 1:** ABO SNPs associated with cardiovascular disease and risk factors.

SNP	Major allele	Minor allele	Position on Ch9 (NCBI Build 137)^a^	Location within *ABO* locus^a^	MAF (NCBI Build 137)^a^	MAF in studied population	Tagged ABO Blood Group	Associated Physiological/ Pathophysiological Trait^b^	Reference
rs558240	G	A	136157133	5′ UTR	0.260	0.39	—	sE-selectin Variance	Qi et al. [21]
						0.38	—	sE-selectin Variance	Paterson et al. [22]
rs495828	G	T	136154867	5′ UTR	0.189	0.261	O	VTE Risk	Heit et al. [23]
						0.17	—	ACE Variance	Chung et al. [24]
rs649129	C	T	136154304	5′ UTR	0.189	—	A1	sICAM-1 Variance	Barbalic et al. [25]
						0.22	—	LDL Variance	Teslovich et al. [26]
rs579459	T	C	136154168	5′ UTR	0.189	—	A1	sICAM-1 Variance	Barbalic et al. [25]
						—	A1	sICAM-1 Variance	Kiechl et al. [27]
						0.2	A1	sE-selectin Variance	Paterson et al. [22]
						—	A1	sE-selectin Variance	Kiechl et al. [27]
						—	A1	sP-selectin Variance	Barbalic et al. [25]
						—	A1	sP-selectin Variance	Kiechl et al. [27]
rs651007	C	T	136153875	5′ UTR	0.190	0.2/0.22	—	VWF Variance	Zabaneh et al. [28]
						—	A1	sICAM-1 Variance	Qi et al. [21]
						—	A1	sICAM-1 Variance	Kiechl et al. [27]
						0.22	A1	sE-selectin Variance	Qi et al. [21]
						0.21	—	Cholesterol Variance	Teslovich et al. [26]
rs630014	G	A	136149722	Intron 1	0.460	0.48/0.48	—	VWF Variance	Zabaneh et al. [28]
						0.48	—	sE-selectin Variance	Paterson et al. [22]
rs529565	T	C	136149500	Intron 1	0.361	—	A11	VTE Risk	Wiggins et al. [29]
						0.37	O/A	MI Risk	Reilly et al. [4]
rs507666	G	A	136149399	Intron 1	0.164	0.2	A1	sICAM-1 Variance	Paré et al. [30]
						0.2	A1	sICAM-1 Variance	Paré et al. [31]
						—	A1	sICAM-1 Variance	Kiechl et al. [27]
rs505922	T	C	136149229	Intron 1	0.348	0.37	O/A	MI Risk	Reilly et al. [4]
						—	—	VTE Risk	Tregouet et al. [32]
rs500498	C	T	136148647	Intron 1	0.481	0.45	—	sICAM-1 Variance	Paré et al. [31]
						0.47	—	sE-selectin Variance	Paterson et al. [22]
rs674302	T	A	136146664	Intron 1	0.376	0.37	O/A	MI Risk	Reilly et al. [4]
rs8176668	A	T	136144059	Intron 1	0.325	0.4	—	sE-selectin Variance	Qi et al. [21]
rs612169	A	G	136143442	Intron 1	0.376	0.37	O/A	MI Risk	Reilly et al. [4]
						—	O	sICAM-1 Variance	Qi et al. [21]
						0.34	O	sE-selectin Variance	Qi et al. [21]
rs545971	C	T	136143372	Intron 1	0.376	0.37	O/A	MI Risk	Reilly et al. [4]
rs643434	G	A	136142355	Intron 1	0.391	0.39	O/A	MI Risk	Reilly et al. [4]
rs644234	T	G	136142217	Intron 1	0.389	0.39	O/A	MI Risk	Reilly et al. [4]
						0.35	—	sE-selectin Variance	Paterson et al. [22]
rs514659	A	C	136142203	Intron 1	0.376	—	O	VWF Variance	Campos et al. [33]
						—	O	VWF Variance	Smith et al. [34]
						0.37	O/A	MI Risk	Reilly et al. [4]
rs8176672	C	T	136142185	Intron 1	0.113	—	B	sE-selectin Variance	Qi et al. [21]
rs2519093	C	T	136141870	Intron 1	0.166	0.235	—	VTE Risk	Heit et al. [23]
rs8176681	T	C	136139754	Intron 1	0.333	0.42	—	sE-selectin Variance	Qi et al. [21]
rs657152	C	A	136139265	Intron 1	0.384	0.34/0.36	O	VWF Variance	Zabaneh et al. [28]
						0.4	O/A	MI Risk	Reilly et al. [4]
						—	A11	VTE Risk	Wiggins et al. [29]
						—	A11	VTE Risk	Tregouet et al. [32]
						0.37	—	sICAM-1 Variance	Paré et al. [31]
						0.38	—	sE-selectin Variance	Qi et al. [21]
						0.35	—	sE-selectin Variance	Paterson et al. [22]
						0.373	O	Phytosterol Variance	Teupser et al. [35]
rs8176694	T	C	136137646	Intron 1	0.116	0.19/0.18	—	VWF Variance	Zabaneh et al. [28]
rs687289	G	A	136137106	Intron 2	0.367	0.37	O/A	MI Risk	Reilly et al. [4]
						0.34	O	sICAM-1Variance	Paré et al. [31]
rs687621	A	G	136137065	Intron 2	0.373	—	—	VWF Variance	Smith et al. [34]
						0.37	O/A	MI Risk	Reilly et al. [4]
						0.34	—	sICAM-1 Variance	Paré et al. [31]
rs688976	C	A	136136770	Exon 3	0.289	0.23/0.22	—	VWF Variance	Zabaneh et al. [28]
rs8176704	G	A	136135552	Intron 3	0.051	—	A2	VWF Variance	Campos et al. [33]
						—	A2	VWF Variance	Smith et al. [34]
						—	A2	VTE Risk	Heit et al. [23]
						—	A2	sICAM-1 Variance	Paré et al. [31]
						—	A2	sICAM-1 Variance	Qi et al. [21]
						—	A2	sICAM-1 Variance	Barbalic et al. [25]
						—	A2	sE-selectin Variance	Qi et al. [21]
						—	A2	sP-selectin Variance	Barbalic et al. [25]
rs549446	C	T	136135238	Exon 4	0.420	0.25/0.24	—	VWF Variance	Zabaneh et al. [28]
rs638756	A	C	136134472	Intron 4	0.300	0.25/0.24	—	VWF Variance	Zabaneh et al. [28]
rs512770	G	A	136133506	Exon 5	0.262	0.21/0.2	O2	VWF Variance	Zabaneh et al. [28]
						—	O2	VWF Variance	Smith et al. [34]
rs8176719	Del	G	136132908	Exon 6	0.348	—	O	VTE Risk	Tregouet et al. [32]
						0.416	O	VTE Risk	Heit et al. [23]
						0.34	O	sE-selectin Variance	Paterson et al. [22]
rs8176722	C	A	136132754	Intron 6	0.131	0.08/0.08	B	VWF Variance	Zabaneh et al. [28]
						—	B	VWF Variance	Tregouet et al. [32]
rs8176734	G	A	136132079	Intron 6	—	—	O12	VTE Risk	Wiggins et al. [29]
rs8176740	A	T	136131472	Exon 7	0.267	0.25/0.24	—	VWF Variance	Zabaneh et al. [28]
rs8176746	G	T	136131322	Exon 7	0.123	—	B	VTE Risk	Tregouet et al. [32]
						—	B	sICAM-1 Variance	Paré et al. [31]
						0.16	—	ACE Variance	Chung et al. [24]
rs8176749	C	T	136131188	Exon 7	0.123	—	B	VWF Variance	Smith et al. [34]
						—	B	VTE Risk	Wiggins et al. [29]
rs8176750	C	Del	136131059	Exon 7	—	—	A2	VTE Risk	Tregouet et al. [32]
rs7857390	G	A	136128546	3′ UTR	0.320	0.4	—	sE-selectin Variance	Qi et al. [21]

ACE: angiotensin-converting enzyme; LDL: low density lipoprotein; MAF: minor allele frequency; MI: myocardial infarction; sICAM-1: soluble intercellular adhesion molecule-1; VTE: venous thromboembolism; VWF: von Willebrand factor.

^a^The chromosomal location and MAF for each SNP table entry was obtained by querying each SNP rs number in the NCBI Single Nucleotide Polymorphism database (dbSNP), build 137 on the 13th of September, 2012 (http://www.ncbi.nlm.nih.gov/projects/SNP/).

^b^The associated physiological/pathophysiological traits were extracted from the National Human Genome Research Institute (NHGRI) GWA catalog database (http://www.genome.gov/gwastudies/) and queried publications from PubMed (http://www.ncbi.nlm.nih.gov/pubmed).
